# Characterization of Cochlear, Vestibular and Cochlear-Vestibular Electrically Evoked Compound Action Potentials in Patients with a Vestibulo-Cochlear Implant

**DOI:** 10.3389/fnins.2017.00645

**Published:** 2017-11-21

**Authors:** T. A. K. Nguyen, Samuel Cavuscens, Maurizio Ranieri, Konrad Schwarz, Nils Guinand, Raymond van de Berg, Thomas van den Boogert, Floor Lucieer, Marc van Hoof, Jean-Philippe Guyot, Herman Kingma, Silvestro Micera, Angelica Perez Fornos

**Affiliations:** ^1^Division of Functional Neurosurgery, Department of Neurology, Inselspital Bern, Bern, Switzerland; ^2^Bertarelli Foundation Chair in Translational Neuroengineering, ÉcolePolytechnique Fédérale de Lausanne, Lausanne, Switzerland; ^3^Service of Otorhinolaryngology and Head and Neck Surgery, Department of Clinical Neurosciences, Geneva University Hospitals, Geneva, Switzerland; ^4^MED-EL, Innsbruck, Austria; ^5^Division of Balance Disorders, Department of Otorhinolaryngology and Head and Neck Surgery, Faculty of Health Medicine and Life Sciences, School for Mental Health and Neuroscience, Maastricht University Medical Center, Maastricht, Netherlands; ^6^International Research Laboratory for Modelling of Physical Processes in Biology and Medicine, Faculty of Physics, Tomsk State University, Tomsk, Russia; ^7^Department of Cardiology, Academic Medical Center, University of Amsterdam, Amsterdam, Netherlands; ^8^Translational Neural Engineering Laboratory, BioRobotics Institute, Scuola Superiore Sant'Anna, Pisa, Italy

**Keywords:** vestibular implant, vestibular prosthesis, neural prosthesis, electrically evoked compound action potential, cochlear implant, bilateral vestibular loss, vestibular function

## Abstract

The peripheral vestibular system is critical for the execution of activities of daily life as it provides movement and orientation information to motor and sensory systems. Patients with bilateral vestibular hypofunction experience a significant decrease in quality of life and have currently no viable treatment option. Vestibular implants could eventually restore vestibular function. Most vestibular implant prototypes to date are modified cochlear implants to fast-track development. These use various objective measurements, such as the electrically evoked compound action potential (eCAP), to supplement behavioral information. We investigated whether eCAPs could be recorded in patients with a vestibulo-cochlear implant. Specifically, eCAPs were successfully recorded for cochlear and vestibular setups, as well as for mixed cochlear-vestibular setups. Similarities and slight differences were found for the recordings of the three setups. These findings demonstrated the feasibility of eCAP recording with a vestibulo-cochlear implant. They could be used in the short term to reduce current spread and avoid activation of non-targeted neurons. More research is warranted to better understand the neural origin of vestibular eCAPs and to utilize them for clinical applications.

## Introduction

The peripheral vestibular organs in the inner ear provide essential information about head motion and orientation. The three semicircular canals and two otolith organs sense rotational and translational movement, and their input contributes to the execution of activities of daily life (e.g., posture, stability, fall prevention, spatial orientation, image stabilization in dynamic situations). Vestibular dysfunction can, for instance, result in dizziness, vertigo and imbalance. Bilateral vestibular hypofunction can significantly reduce quality of life (Gillespie and Minor, 1999; Guinand et al., 2012) and it is estimated that 500,000 patients are affected in the United States and Europe (Ward et al., 2013). Current clinical treatments based on medication and rehabilitation remain inadequate (Sun et al., 2014).

To restore vestibular function, several research groups have been developing vestibular implants and have provided proof-of-concept studies in animal models and human patients (Fridman and Della Santina, 2012; Merfeld and Lewis, 2012; Guinand et al., 2015; Phillips et al., 2015). Most groups have used cochlear implants as a platform technology to accelerate development and tap the implants' existing body of research and expertise. These implants restore auditory function and are to date the most successful neuroprosthesis with more than 600,000 implantations worldwide (see e.g., http://www.medel.com/cochlear-implants-facts/; http://www.earfoundation.org.uk/hearing-technologies/cochlear-implants/cochlear-implant-information-sheet). Our group has developed an original concept of using a combined device incorporating an array to be implanted in the cochlea as well as independent electrode branches for the three semicircular canals to activate the vestibular afferents. To date, 13 patients suffering from both hearing loss and bilateral vestibular hypofunction have been implanted with such a device (Guinand et al., 2015).

In cochlear implants, various objective measures exist to complement behavioral measures and to assess, for example, electrode placement or implant performance (Shallop, 1993). In particular, the electrically evoked compound action potential (eCAP) is an electrophysiological measurement that can be carried out relatively fast and easy in most modern cochlear implants with a two-way telemetry system (Hughes, 2012). Its signal has a double peak complex and reflects the collective response of numerous fibers in the target nerve. It is believed to be composed of axonal and dendritic components (Stypulkowski and Van den Honert, 1984; Lai and Dillier, 2000). In clinical practice eCAPs have proven to be a useful tool in specific patient populations. For example, this technique can be used by audiologists to program cochlear implants in infants (Gordon et al., 2004). Recent eCAP research has proposed advanced recording techniques and analyses to better understand neural activation and the electrode-neuron interface (Cosentino et al., 2016; DeVries et al., 2016).

In vestibular implants, eCAP can improve electrode placement intraoperatively and add supplemental information about stimulation effects (Nie et al., 2011; Phillips et al., 2015). We have previously characterized eCAPs in guinea pigs and correlated it with eye movement responses (Nguyen et al., 2013). In the long-term, eCAPs could potentially serve as a fast feedback signal for an adaptive vestibular implant (DiGiovanna et al., 2010). In the present study, we attempted first eCAP recordings with a vestibulo-cochlear implant in patients. This design only provides a single electrode per semicircular canal, while eCAP recordings classically require one stimulating and one recording electrode. Therefore, it is necessary to investigate other non-classical setups to explore the possibility for eCAP recordings with our device. Specifically, we acutely recorded eCAPs to three different setups: (i) cochlear setup (stimulating and recording electrodes in the cochlea), (ii) cochlear-vestibular or mixed setup (stimulating electrode in the cochlea, recording electrode in the ampulla of a semicircular canal and vice versa), and (iii) vestibular or trans-canal setup (stimulating electrode in one canal, recording electrode in another canal). Particularly the third setup would provide different insights from previously presented *intra*-canal eCAPs (Nie et al., 2011) where one stimulating and a second recording electrode are placed in the same semicircular canal.

We recorded eCAPs in four patients for all setups (i–iii) and observed slight differences in morphology, magnitude and latency. In one subject, we additionally measured eCAPs during continuous stimulation and found a change in magnitude over time. These findings revealed interesting eCAP characteristics and warrant further research to evaluate a possible clinical use for programming vestibular implants.

## Materials and methods

### Patients, implant and surgery

Experiments were performed in accordance with the Declaration of Helsinki. The ethics committees of the University Hospitals Geneva (NAC 11-080) and the Maastricht University Medical Center (NL36777.068.11/METC 11-2-031) approved this study for which four patients were available. Patients provided written prior consent. These are named Subject 1 through Subject 4 here. All subjects suffered from hearing loss and bilateral vestibular hypofunction before implantation. Subject 1 suffered from a trauma and the implantation was performed at age 67 in the left ear. Subject 2 had meningitis and received the implant in the right ear at age 48. Both subjects 3 and 4 suffered from DFNA9, an autosomal dominant nonsyndromic congenital disease (Khetarpal, 2000), and received the implant at ages 66 and 67 in the left ear. All subjects were recruited at the Service of Otolaryngology, Head and Neck Surgery at the Geneva University Hospitals and at the Division of Balance Disorders at the Maastricht University Medical Center. Inclusion criteria, the modified cochlear-implant and surgical procedures were described in detail elsewhere (Perez Fornos et al., 2014; Guinand et al., 2015; Van de Berg et al., 2015).

Briefly, cochlear implants (MED-EL, Innsbruck, Austria) were modified to provide nine electrode contacts for cochlear stimulation (numbered 1–9) and one vestibular electrode contact for each of the three semicircular canals (contact 10 for the lateral semicircular canal, contact 11 for the superior semicircular canal and contact 12 for the posterior semicircular canal). The latter three contacts were placed intra-labyrinthine in proximity to the ampulla of the corresponding canal.

### Experimental paradigms

We performed different experiments to test the feasibility of eCAP recordings with the given vestibulo-cochlear implant and, if feasible, to characterize the responses to acute stimulation and continuous stimulation. Experiment A studied responses to single pulses during acute stimulation, whereas experiments B1 and B2 studied responses during continuous stimulation.

In experiment A, we investigated the feasibility of recording eCAPs with a vestibulo-cochlear implant and if feasible, to characterize these eCAPs with amplitude growth functions. Toward this, eCAPs were recorded in subjects 1 to 4 during acute testing sessions of approximately 1 h. They were recorded in response to single stimulation pulses and no other stimulation was applied between recordings. Firstly, the impedance of all electrode contacts was measured with MED-EL's MAESTRO software to confirm electric functioning and determine the maximal current amplitude that stayed within the compliance voltage and charge injection limits. Due to slightly different electrode impedances, this maximal amplitude was subject-specific and was applied for all stimulation contacts for consistency and better comparison. Secondly, a sweep was performed to test all possible combinations of stimulation and recording pairs. These sweeps were performed with only four current amplitudes up to the maximal current amplitude and minimal number of iterations to identify suitable electrode pairs for more detailed recordings later.

eCAPs are typically recorded by stimulating with one electrode and recording from another adjacent or near electrode. However, given the design of the present vestibulo-cochlear implant with only a single electrode per semicircular canal, we initially evaluated whether it was possible to achieve single-electrode eCAP recordings (i.e., use the same electrode for stimulation and recording). Unfortunately this was not feasible despite trying a broad range of stimulation parameters as well as an alternative stimulation paradigm with triphasic pulses (Bahmer and Baumann, 2012). We therefore resorted to typical stimulation-recording pairs. For instance, contact 1 could be used for stimulation and the adjacent contact 2 for recording. This would be denoted as *S1R2* in this study. Combinations of stimulation and recording electrodes that showed a single or double peak complex (negative or positive peak, or N-P) were then selected for further characterization. Thirdly, the amplitude growth function (AGF, eCAP responses to increasing current amplitudes) was recorded for the selected combinations to 12 current amplitudes up to the maximal current amplitude for the given subject.

The stimulation-recording pairs can be divided into three different categories: (i) a **cochlear setup**, where stimulation and recording electrode were in the cochlear array; (ii) a cochlear-vestibular or **mixed setup**, where the stimulation electrode was in the cochlear array and the recording electrode in a vestibular branch and in the mirrored condition, that is the stimulation electrode in a vestibular branch and the recording electrode placed in the cochlear; and (iii) a vestibular or **trans-canal setup**, where the stimulation electrode was in one vestibular branch and the recording in another vestibular branch. Setup (iii) was also performed in the opposite direction similar to setup (ii). These electrode pairs and the mirrored conditions were then analyzed separately. Information about subjects and setups are summarized in Table 1. Stimulation consisted of biphasic pulses with a phase width of 50 μs and an inter-phase gap of 2.1 μs. eCAP recording was performed with an alternating polarity paradigm to reduce artifact: The response to a cathodic-anodic pulse was combined with the response to an anodic-cathodic pulse of the same current amplitude (Miller et al., 2000). To improve signal-to-noise ratio, recordings were averaged over 15 repetitions for sweeps and 16 repetitions for amplitude growth function recordings. These repetitions were presented every 100 ms with sufficient time for eCAP responses to recover to baseline.

**Table 1:** Summary of subjects and setups.

**Patient**	**Etiology**	**Cochlear setup**	**Mixed setup**	**Trans-canal setup**	**Max. amplitude [cu]**
1	Trauma	S1R2	S9R11/S11R9	S10R11/S11R10	900
2	Meningitis	S2R3	S6R11/S11R6	S11R12/S12R11	500
3	DFNA9	S1R2	S8R11/S11R8	S10R11/S11R10	700
4	DFNA9	S8R9	S8R10/S10R8	S10R12/S12R10	900

*Electrode pairs for the mixed and trans-canal setups were recorded also in mirrored conditions, e.g., eCAPs for patient 1 were recorded for S10R11 (stimulation with electrode 10 and recording with electrode 11) and S11R10. The maximum amplitude was applied across all three setups for consistency*.

In experiments B1 and B2, we investigated the effect of continuous stimulation on eCAP responses and whether eCAP responses are subject to adaptation as seen for vestibular-ocular reflex responses (Guyot et al., 2011). This condition was designed to represent real-life functioning of the vestibular implant, where continuous stimulation would be required to encode head motion in both excitatory and inhibitory directions (Merfeld et al., 2007). Subject 1, who was available for these additional experiments, was exposed to 50 min of continuous stimulation at 400 pulses per second (pps) with phase widths of 200 μs and a current amplitude of 400 current units (cu, 1 cu ~1 μA). This current amplitude was chosen at slightly < 50% of the maximal current amplitude for this subject (900 cu) and is a typically used amplitude for continuous or steady-state stimulation (Perez Fornos et al., 2014). At 10 min intervals, eCAP responses were recorded in response to stimulation pulses with the same current amplitude of 400 cu, but with a phase width of 50 μs to avoid signal saturation (experiment B1, investigating adaptation in eCAP responses to steady-state stimulation). In experiment B2, eCAP responses were recorded also at 10 min intervals, but to stimulation pulses with a current amplitude of 900 cu and 50 μs phase width. This higher current amplitude was chosen to investigate whether eCAP responses showed adaptation to stimulation pulses with current amplitudes higher than steady-state stimulation.

### Stimulation, recording, and analysis

All stimulation and recordings for experiments A and B were applied with a MED-EL Research Interface Box 2 (Bahmer et al., 2010) and programmed through custom scripts in MATLAB 2011b (MathWorks, Natick, MA, USA). Recordings started after a blanking delay of 170 μs after stimulation onset to avoid stimulation artifact. Recordings consisted of 2048 samples gathered at 1.2 MHz (a recording window of ca. 1.7 ms). An analog-digital conversion was performed by the implant and interface box before the recordings were saved and post-processed in MATLAB. In post-processing, recordings were averaged across 60 iterations. A 4th-order digital Butterworth low-pass filter with 5 kHz cut-off frequency was applied to the result to further reduce noise. To detect negative (N) and positive (P) peaks, a 14th-order polynomial was fitted to the filtered recording and local optima (i.e., negative and positive peaks) were computed with the MATLAB command *polyder* to calculate the derivative of the polynomial and the command *roots* to determine the optima of the polynomial. Most relevant were the first negative peak and the first positive peak that were then used to calculate the N-P voltage (MATLAB script provided by the MED-EL's Research Interface Box 2).

To complement analysis, we measured the distances between electrodes and ampullae. Computed tomographic (CT) images were collected using a conventional high-resolution CT of the mastoid (Subject 1) or by using cone beam CT (subjects 2–4) as outlined elsewhere (Dees et al., 2016). Image resolution varied between 0.15 and 0.4 mm. Additionally, pre-implant MRIs (Subjects 3, 4) or CT's (Subjects 1, 2) of the inner ear were retrieved from these subjects and superimposed after registration with post-implant CT to localize both the electrode and ampulla using previously validated techniques (Fedorov et al., 2012; Dees et al., 2016). Measurements were performed in 3DSlicer (The Slicer Community, https://www.slicer.org) using fiducials. Fiducial placement was repeated 15 times to determine an accurate position in three-dimensional space and the distance was calculated as the average of these 15 measurements. Statistical analyses of these were performed in Mathematica (Version 10.4, Wolfram Research, Inc., Champaign, Il, USA, 2016).

## Results

### Experiment A: amplitude growth functions for three setups

In experiment A, we recorded eCAPs and AGFs for cochlear, mixed and trans-canal setups. The maximal current amplitude was 900 cu for subject 1,500 cu for subject 2,700 cu for subject 3, and 900 cu for subject 4. The sweeps provided pairs of stimulation and recording electrodes where eCAPs displayed a single or double peak complex that were then characterized further.

**Figure 1:**
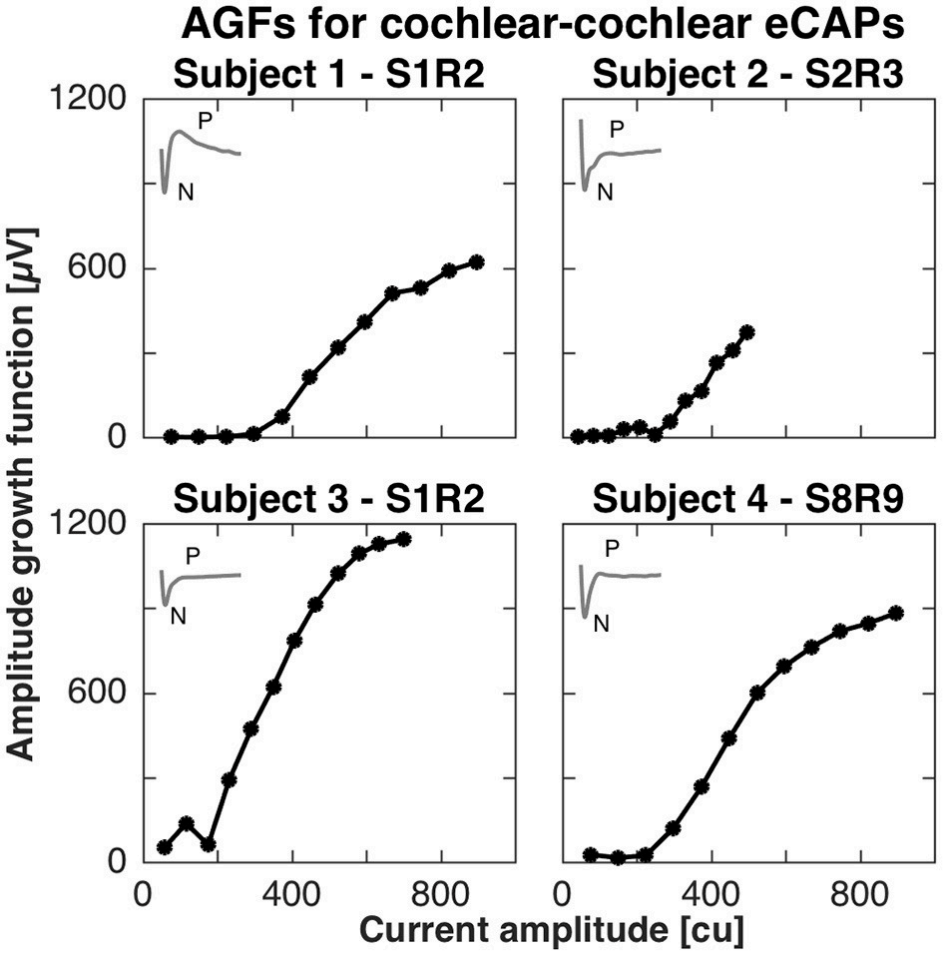
Amplitude growth functions for the cochlear setup in subjects 1–4. The gray inset in the top left corner of every panel illustrate the eCAP response to the highest current amplitude in that subject. The AGFs were measured for 12 different current amplitudes in each subject and were recorded with apical electrode contacts in subjects 1–3 and basal electrodes for subject 4. A clear N-P complex was best visible in the eCAP response of subject 1, while the slope of the AGF was largest in subject 3 (2.3 μV/cu).

For the cochlear setup, we measured the AGFs with apical electrodes (contacts 1–3) in subjects 1, 2, and 3. Specifically in subject 1, we stimulated with electrode 1 and recorded with electrode 2 (denoted S1R2), in subject 2 we stimulated with electrode 2 and recorded with electrode 3 (denoted S2R3), and in subject 3 we stimulated with electrode 1 and recorded with electrode 2 (denoted S1R2). In subject 4, eCAPs could not be obtained with apical electrodes, but was successful with the basal electrode contacts 8 and 9 (denoted S8R9). All AGFs are illustrated in Figure 1. eCAPs in subject 1 had the most pronounced double peak N-P complex of all subjects, whereas the other subjects had visible N peaks, but less pronounced P peaks. The insets in Figure 1 show the eCAP recording for the highest current amplitude. The AGFs displayed typical sigmoid shapes with a flat portion for low current amplitudes and a rising portion for further increasing current amplitudes. The rising portion was steepest in subject 3, where the AGF increased by 1150 μV over ca. 500 cu or with a slope of 2.3 μV/cu. The slopes for subjects 2, 3, and 4 were 1.0, 1.5 and 1.4 μV/cu, respectively, assuming a linear fit for the rising portion.

**Figure 2:**
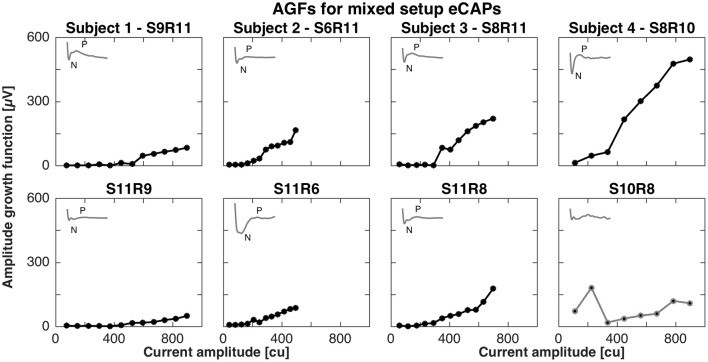
Amplitude growth functions for the mixed setup in subjects 1–4. The gray insets in the top left corner of each panel represent the eCAP response to the largest current amplitude. eCAPs and AGFs were measured with a stimulating electrode in the cochlea and a recording electrode in a semicircular canal **(Top row)** and mirrored conditions **(Bottom row)**. A clear N-P complex was only observed in subjects 3 and 4 (S8R11 and S8R10, respectively). Slopes of the AGFs were generally smaller than for the cochlear setup (between 0.1 and 0.8 μV/cu for mixed setup, between 1.0 and 2.3 μV/cu for cochlear setup). S10R8 for subject 4 was grayed out here and in following plots as the eCAP response was not likely to be a neuronal response.

For the mixed setup, eCAPs were successfully recorded between middle (contacts 4–6) or basal electrodes (contacts 7–9) and vestibular electrodes. We were not able to record eCAPs between apical (contacts 1–3) and vestibular electrodes (contacts 11–12). The pairs of successful stimulation and recording electrodes were S9R11, S6R11, S8R11, and S8R10 and mirrored conditions for subjects 1, 2, 3, and 4, respectively. Figure 2 shows the AGFs for these pairs. Amplitude growth functions for the mixed setup also exhibited a flat and rising portion. However, the rising portions for mixed setups were markedly less steep than for the cochlear setup and ranged from 0.2 to 0.8 μV/cu when the stimulating electrode was in the cochlea and 0.1 to 0.4 μV/cu when the stimulating electrode was in a semicircular canal. The insets in Figure 2 illustrate the eCAP recording for the highest current amplitude and underline the difference in morphology compared to the cochlear setup. A double peak complex was detectable when stimulating the cochlea, but the complex was less visible for the opposite condition. Note that we excluded S10R8 from subject 4 and removed it from further analysis as it did not display a clear peak complex.

**Figure 3:**
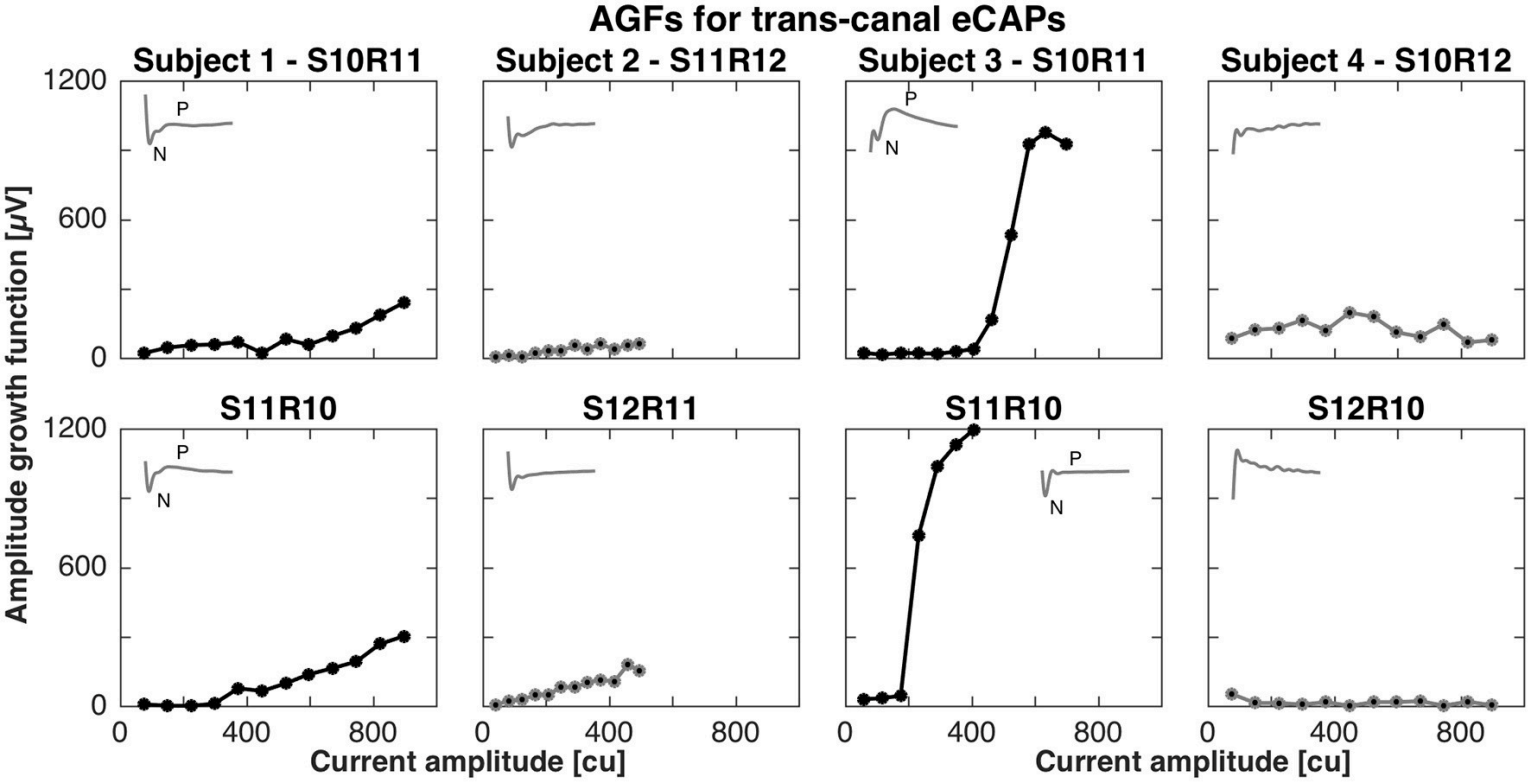
Amplitude growth functions for the trans-canal setup in subjects 1–4. The gray insets in the top left corner of each panel represent the eCAP response to the largest current amplitude. eCAPs and AGFs were measured with a stimulation electrode in one semicircular canal and the recording electrode in another canal. A clear N-P complex could be observed only in subjects 1 and 3. Subject 3 had the largest N-P magnitudes for this setup and the corresponding slope was 4.1 μV/cu. In the case of subject 1, the N-P complex was less pronounced and the AGF slope was 0.6 μV/cu. eCAPs and AGFs for both subjects 2 and 4 were grayed out from here, as the responses were unlikely of neuronal origin, but rather a result of stimulation artifact.

For the trans-canal setup, the pairs of stimulation and recording electrodes were S10R11, S11R12, S10R11, and S10R12 for subjects 1 to 4, respectively. Figure 3 depicts the AGFs for these pairs, in both stimulation-recording directions. A clear N-P complex could only be observed in the responses recorded in subjects 1 and 3, and the corresponding AGFs had distinct flat and rising portions with slopes of 0.6 and 4.1 μV/cu, respectively. The trans-canal eCAP responses for subjects 2 and 4 did not show a typical N-P peak complex and additionally recorded recovery functions indicated artifact. Therefore, responses for subjects 2 and 4 were grayed out and excluded from further analysis (see insets of Figure 3).

**Figure 4:**
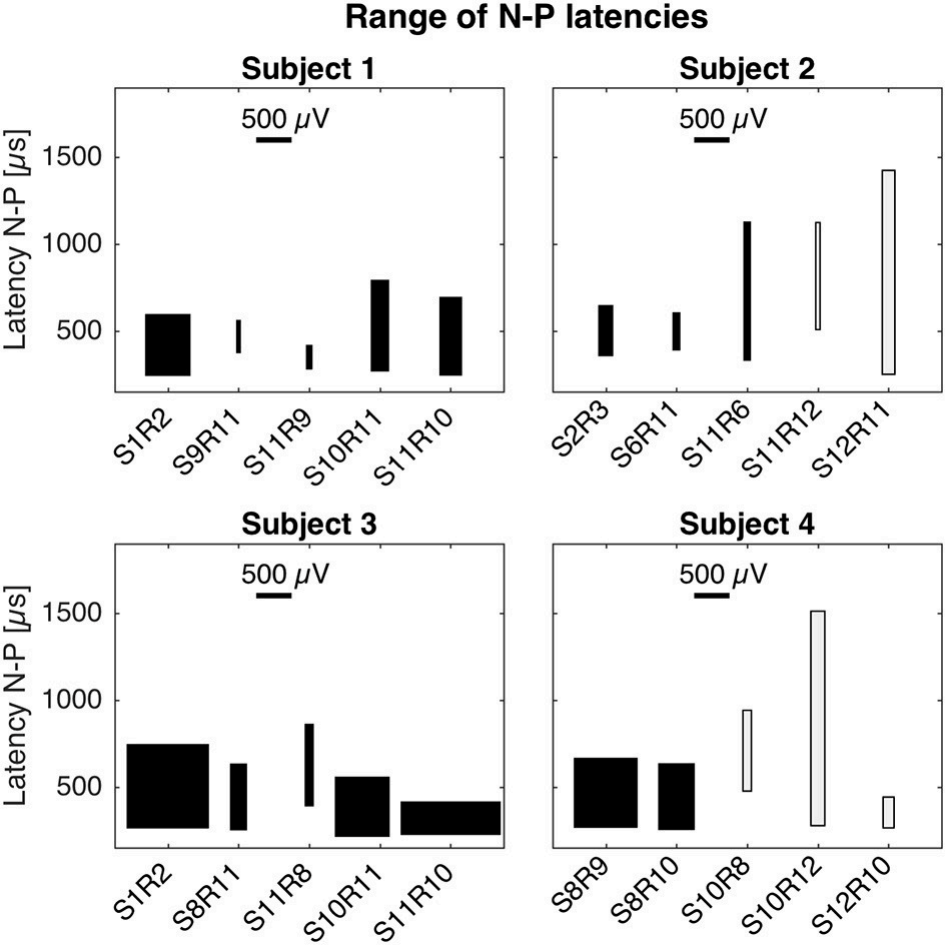
Magnitudes and latencies of eCAPs in all setups. For a given setup, the box captures the range of N-P magnitudes (width of box) and the range of N-P latencies (height of box). The bottom of the box represents the earliest N peak, while the top of the box represents the latest P peak. These values were taken from the averaged eCAP responses across all current amplitudes (cf. Figures 1–3). Latencies of N-P peaks were comparable across setups and patients. N peaks occurred around 250 μs after stimulation onset and P peaks occurred between 600 and 850 μs. N-P magnitudes denoted by the width of the black boxes were similar for cochlear and trans-canal setups, whereas magnitudes for mixed setup were smaller (cf. subjects 1 and 3). Pairs for subjects 2 and 4 were grayed out as they were likely the result of stimulation artifact.

Latencies and magnitudes of eCAP responses are illustrated in Figure 4. N-P latencies were comparable across all setups. Negative peaks occurred at approximately 250 μs after stimulation onset and positive peaks between 600 and 850 μs. Exceptions were S11R9 in subject 1 with a P latency at 420 μs and S11R6 in subject 2 with P latency at 1,127 μs. Magnitudes of the N-P voltages were generally larger for the cochlear setup, shown as widths of the boxes in Figure 4.

**Figure 5:**
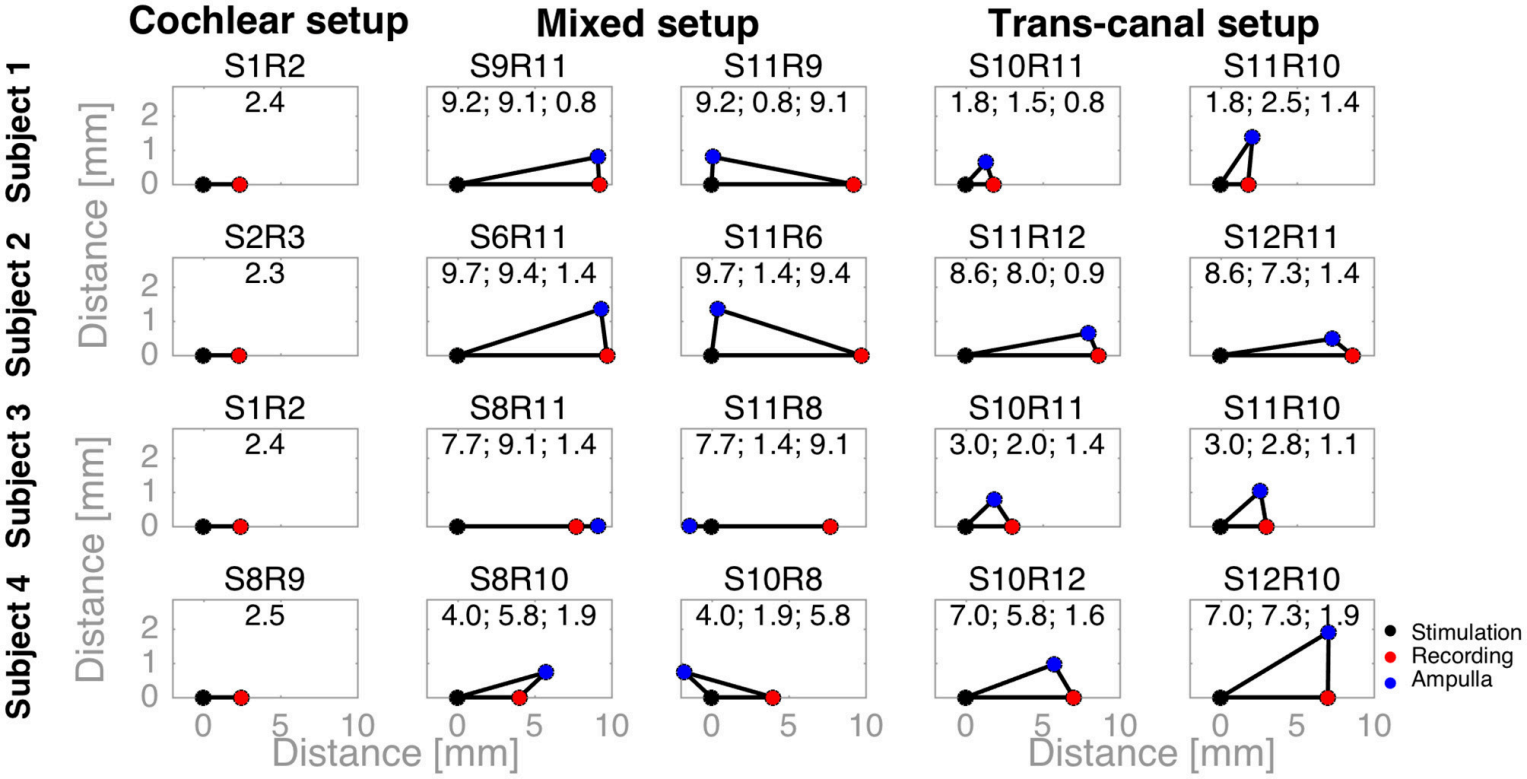
Distances between stimulation, recording electrodes and ampullae illustrated as triangles. A black dot denotes the stimulation electrode, a red dot the recording electrode, and a blue dot the ampulla of the corresponding vestibular electrode (e.g., the lateral ampulla for electrode 10). For cochlear setups, ampullae were not applicable, whereas for trans-canal setups the ampulla to the corresponding *recording* vestibular electrode was used (e.g., the superior ampulla for a recording electrode 11). The text above each triangle notes the electrode pair tested. The values are the distances in millimeters. First, the distance between the stimulation and recording electrodes; second, between the stimulation electrode and the ampulla; and third, between the recording electrode and ampulla.

**Figure 6:**
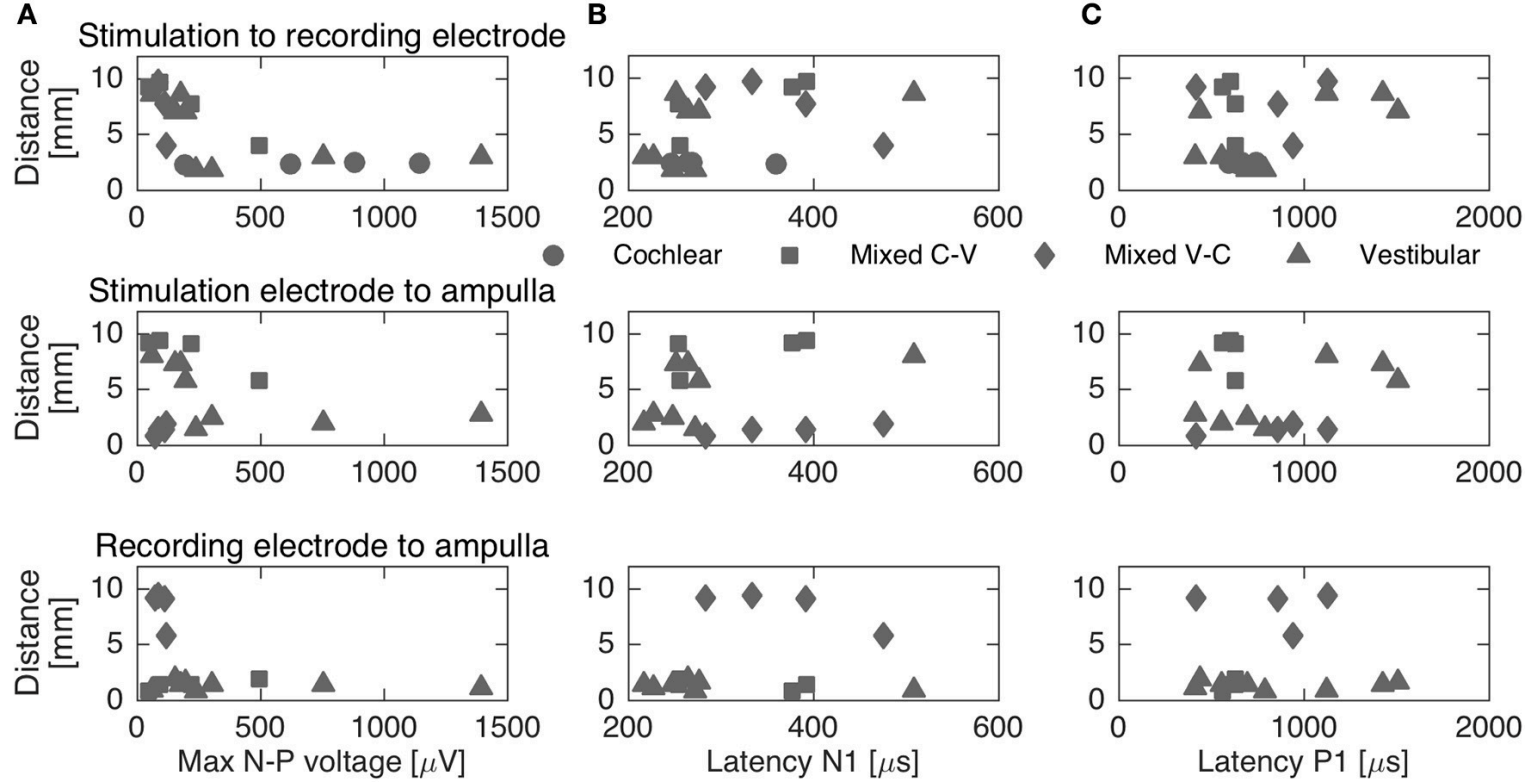
Distances between stimulation, recording electrodes and ampullae vs. maximum N-P voltage **(A)** and N-P latencies (**B**,**C**, respectively). The different setups are represented through different markers. No clear correlation could be established between distances and N-P voltages or latencies.

To complement the latency analysis, distances between stimulation and recording electrodes were illustrated in Figure 5. Distances between adjacent cochlear electrodes in the cochlear setup were shortest with about 2.4 mm. Larger distances were measured for the mixed setups, for instance up to 9.7 mm for S6R11 in subject 2. In the trans-canal setup, distances were generally short between electrodes 10 and 11 (< 3 mm, subjects 1 and 3). But they were larger, as expected, when the posterior electrode 12 was involved (up to 8 mm in subjects 2 and 4). Figure 6 additionally explores the impact of the distance between stimulating and recording electrodes to the ampulla. No clear correlations could be observed between N-P voltages (Figure 6A) or N and P latencies (respectively Figures 6B,C) and these distances. The only interesting observation that deserves to be highlighted is that it appears that the stimulation-to-recording electrode and recording-electrode-to-ampulla distance should be shorter than 5 mm in order to increase the probability of eliciting an eCAP with an amplitude larger than 500 μA. However, this short distance does not seem to be the main determining factor.

**Figure 7:**
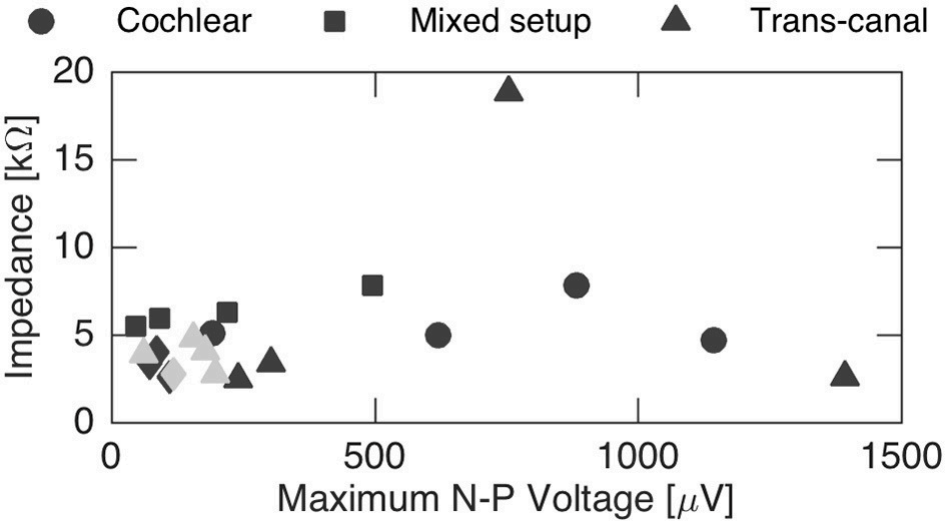
Influence of impedance on the maximum N-P voltage. No general impact could be inferred from the recordings. Gray data points represent discarded sets of subjects 2 and 4 that were likely influenced by stimulation artifact.

Finally, N-P magnitudes were plotted against electrode impedance in Figure 7. We did not observe a clear dependence between electrode impedances and the N-P magnitudes. No linear or polynomial dependence could be found for the N-P magnitudes of the different setups with respect to impedance.

### Experiment B1: eCAP responses obtained during continuous stimulation (same amplitude eCAP stimulation)

**Figure 8:**
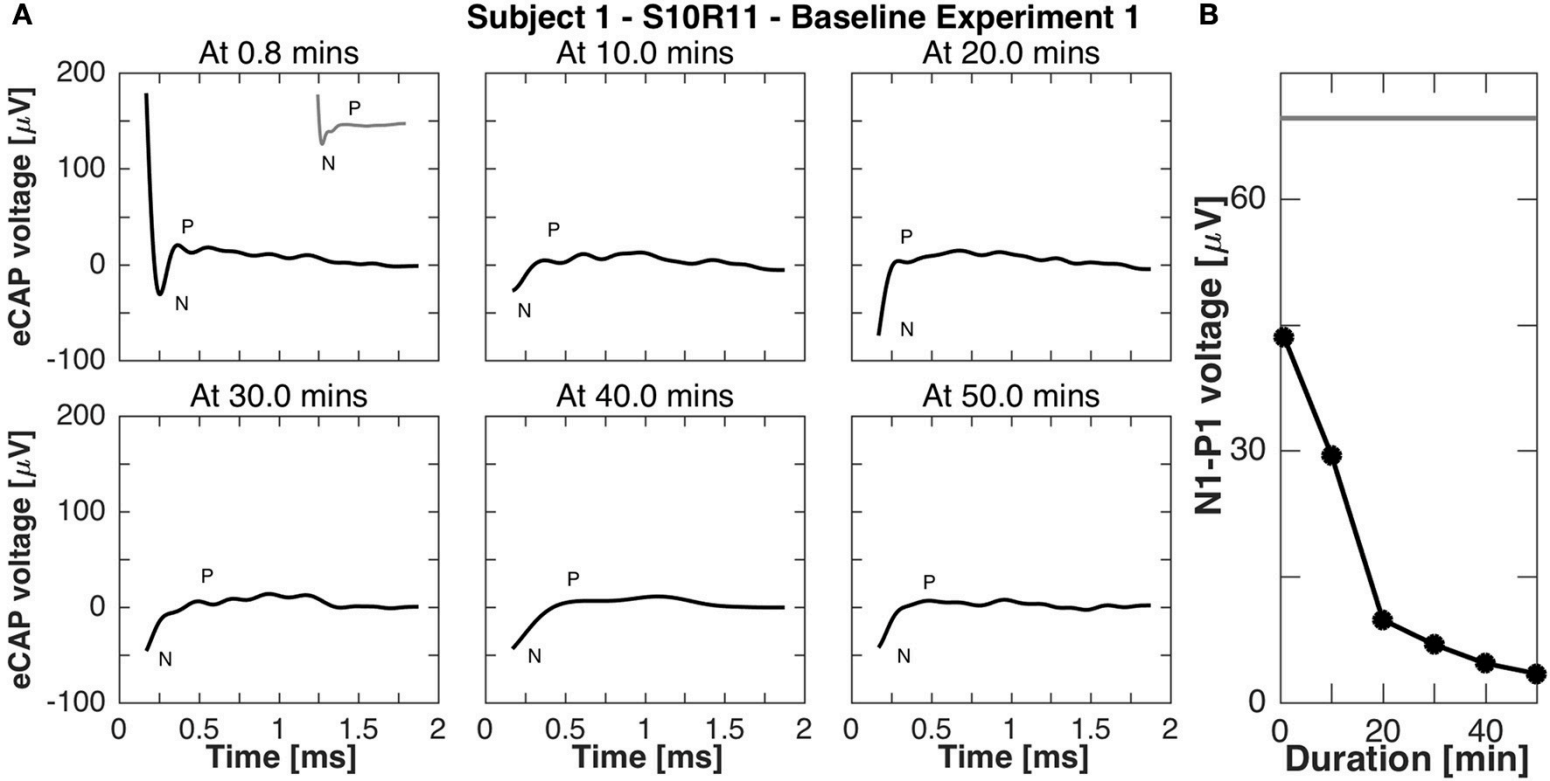
eCAP responses during continuous stimulation of 50 min in subject 1 (experiment B1). **(A)** eCAP was recorded at 10 min intervals in response to a pulse with the *same* current amplitude as the continuous stimulation (but a shorter phase-width, 50 vs. 200 μs, average of 60 iterations). Only at 0.8 min did the eCAP response have a similar morphology to that observed in experiment A *without continuous* stimulation (gray inset at 0.8 min for comparison). At that time point the N peak was still clearly visible, but for later time points only a part of it was visible. **(B)** The N-P amplitude measured in this experiment was smaller than that measured experiment A *without continuous* stimulation at all time points, the eCAP responses here had a smaller N-P amplitude. The gray horizontal line was the N-P magnitude from experiment A for this current amplitude.

Subject 1 was available for further recordings to study eCAP responses during continuous stimulation. In experiment B1, eCAP responses for the vestibular setup S10R11 were recorded every 10 min during 50-min stimulation. Importantly, responses were recorded to stimulation pulses of the *same current amplitude* as the continuous stimulation, but shorter phase width (50 μs phase width compared to 200 μs to avoid signal saturation). The results of this experiment are presented in Figure 8. Rapidly (i.e., after < 1 min of stimulation) the N-P peak complex became less pronounced and the N-P magnitude decreased to 4 μV. The N-P peak complex practically disappeared for all subsequent measurements (10, 20, 30, 40, and 50 min after baseline stimulation onset).

### Experiment B2: eCAP responses obtained during continuous stimulation (higher amplitude eCAP stimulation)

**Figure 9:**
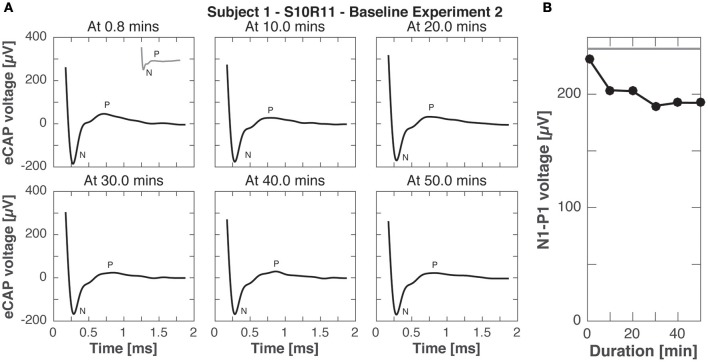
eCAP responses during continuous stimulation of 50 min in subject 1 (experiment B2). **(A)** eCAP was recorded in response to a pulse with the *maximal* current amplitude at 10 min intervals (average of 60 iterations). No significant difference in morphology was observed with increasing duration of stimulation (gray inset at 0.8 min from experiment A for comparison). **(B)** N-P magnitudes were smaller at all time points than for the same pulse *without continuous stimulation* in experiment A (gray horizontal line).

Experiment B2 was like experiment B1, but eCAP responses were recorded to stimulation pulses with *higher current amplitude* (900 cu compared to 400 cu for continuous stimulation). Figure 9 shows the eCAP responses, N-P magnitude did not change significantly over time, only slightly decreasing from the that obtained in experiment A (200 vs. 240 μV for the same electrode pair setup and current amplitude, compare Figures 3, 8).

## Discussion

In the present study, we recorded eCAPs with a combined cochlear-vestibular implant in four patients. eCAPs were measured in two experiments. In experiment A, eCAPs were recorded to single stimulation pulses. In the second experiment B, eCAPs were recorded during continuous stimulation that would mimic chronic use of a vestibular implant.

### eCAP characteristics and neuronal origin

In experiment A, we characterized eCAPs and AGFs for cochlear, mixed and trans-canal setups. In terms of magnitudes, we expected to observe more pronounced N-P peaks and larger magnitudes for the cochlear setup, since both stimulation and recording electrodes would be close and in the same structure, i.e., in the cochlea. Indeed, eCAP peaks were more evident for the cochlear setup (where distances between electrodes were ca. 2.4 mm) than for the mixed setup (where distances were larger between 4.4 and 9.7 mm). For the trans-canal setup eCAP magnitudes were similar to the cochlear setup for subject 3, but slightly smaller for subject 1, while stimulation-to-recording electrode were comparable for the cochlear and trans-canal setups (2.0 mm). Data illustrated in Figures 5, 6 give an additional hint of the complex interplay of N-P magnitudes and distances between electrodes as well as distances between electrodes and ampullae. From this figure it is clear that distance is not the only determining factor. In addition, our experimental protocol was not optimized to explore this particular issue. Therefore, this interesting issue remains to be addressed with a more specific stimulation paradigm in a follow-up study.

Electrode impedances, on the other hand, did not show a clear influence on N-P magnitudes. This is probably due in great part to the impedance recording conditions, where measurements with the clinically approved MAESTRO system by MED-EL were possible only between each electrode and the implant case, but not directly between them. Therefore, to highlight the specific influence inter-electrode impedances in the amplitude of the eCAP response other, more specific impedance measurements might be required and could be implemented with a research interface box extension.

We observed morphological differences in the eCAP recordings across subjects and across setups. For the cochlear setup, a double peak complex with N and P peaks was only observed in subject 1. For the mixed and trans-canal setups, double peak complexes were observed in subjects 1, 3, and 4. All other electrode pairs showed an N peak only, this was notably the case for subject 2 across all setups. These differences in shape could indicate differences in neuronal survival (Stypulkowski and Van den Honert, 1984; Lai and Dillier, 2000). In previous studies with these subjects, we reported activation of the vestibular-ocular reflex through vestibular stimulation (Perez Fornos et al., 2014; Guinand et al., 2015; Nguyen et al., 2016). Peripheral vestibular neurons should be therefore present. Stypulkowski and Van den Honert (1984) recorded *directly* from the auditory nerve in the cat and hypothesized that the earlier of their two peaks was the response of axons proximal to the soma, while the later peak represented activation of the dendrites. Given the placement of the electrodes in the cochlea and the ampullae of the semicircular canals here in our subjects, we would assume that in our recordings the first N peak was a response from dendrites of the ganglion cells and the later P peak, if existing, from the more distant axons. However, this is a very cautious interpretation of our data and warrants more extensive research to identify the neuronal origin of mixed and trans-canal eCAPs.

Latencies for the N-P peaks were similar across the three different setups. N peaks were observed around 250 μs after stimulation onset, and P peaks around 600–850 μs. These values are consistent with cochlear implant literature (Abbas et al., 1999), and also with eCAPs recordings from an *intra*-canal setup, where stimulation and recording electrodes were placed in the same semicircular canal (Nie et al., 2011). Considering the distances between electrodes (cf. Figures 5, 6) and the findings from above cited studies, we believe that recorded eCAPs represent neuronal activation close to the recording electrode rather than a neuronal response traveling from the stimulating to the recording electrode. In other words, if the observed responses corresponded to a neuronal response traveling from the stimulating to the recording electrode, latencies would have been markedly different for mixed setup recordings (where distances were about four times the distances of cochlear or trans-canal setup).

Adaptation of eCAP responses were studied with experiments B1 and B2 as responses were recorded during continuous stimulation. The findings highlight the fast adaptation in the peripheral vestibular system as shown in Figures 8, 9 with decreasing N-P amplitudes over time. In fact, the N-P voltage was already smaller than the acute responses in experiment A after only 0.8 min of stimulation. This is in line with our previous study demonstrating adaptation of vestibular-ocular reflex responses to continuous stimulation within 30 min of initial implant activation. That adaptation time further accelerated with successive on-off switching of stimulation (Guyot et al., 2011). eCAPs could be therefore used as additional metric to study sites of adaptation and plasticity in the peripheral and central vestibular system (DiGiovanna et al., 2016; Mitchell et al., 2016).

### Limitations and further improvements

The current version of our cochlear-vestibular setup was limited to trans-canal eCAP recordings, since only one electrode contact was inserted per semicircular canal. More contacts per canal would allow for intra-canal eCAP recording, that could be clinically useful in the future. For example, intra-canal eCAPs could be used to maximize activation of targeted neurons, while keeping current spread to non-targeted neurons to a minimum through measuring trans-canal eCAPs. Indeed, Phillips et al. (2015) had an implant design allowing for intra-canal eCAP recording and used it to guide electrode placement during surgery and monitor post-operative activation of afferent fibers. But they did not report any trans-canal eCAPs.

Regarding signal analysis, further improvements could include more elaborate recording paradigms and analysis techniques for small N-P magnitudes, such as independent component analysis to reduce artifact (Akhoun et al., 2013). Extensions of computational models for vestibular implants could also provide further insights about eCAP recordings (Hayden et al., 2011; Marianelli et al., 2015).

Other limitations deserve to be mentioned. Four patients were studied herein and responses between subjects showed a marked variability, which may be in large part due to differences in neural survival and electrode placement. Regarding the latter, eCAPs could provide helpful information as reported by DeVries et al. (2016). DeVries et al. that found correlations between eCAP features and the quality of the electrode-neuron interface in adult cochlear implant users. Prado-Guitierrez et al. (2006) studied changes in cochlear eCAP responses in a guinea pig model and found reductions in N-P magnitude with increasing neural loss. Therefore, a better understanding of these factors through eCAP recordings could then be leveraged during surgery to adapt electrode placement and address inter-patient variability.

Another noteworthy limitation are the short phase durations of the stimulation pulses used herein. The most effective vestibular stimulation paradigm observed for our vestibulo-cochlear implant consists of biphasic pulses with phase widths ranging between 200 and 400 μs (Perez Fornos et al., 2014; Guinand et al., 2015). However, such large phase widths cannot be used during eCAP recordings since they lead to amplifier saturation (unpublished observation from our group). In experiments B1 and B2, we applied typical continuous stimulation with phase widths of 200 μs and only reduced the phase width to 50 μs during eCAP recording. This was well tolerated by the subject, but of course only partly replicates the effect of adaptation of the vestibular pathways to continuous stimulation.

### Potential applications of cochlear-vestibular and vestibular eCAPs

Our findings suggest that eCAP recordings with a vestibulo-cochlear implant could be used to reduce current spread and thus erroneous activation of non-target neurons by primarily guiding electrode placement during surgery. Specifically, the mixed setup recordings can be utilized to reduce auditory sensations when stimulating a vestibular electrode. Such a sound percept was previously reported by subject 3 for stimulation of contacts 10 and 11 (listed as subject 10 in Guinand et al., 2015). Trans-canal eCAPs can be used to reduce current spread between different semicircular canals and thus improve behavioral responses, such as eye movement responses post-surgery (Nie et al., 2011).

eCAP recordings with a planned refined implant, that would feature more than one electrodes contact per semicircular canal, could be used not only during surgery, but also postoperatively. The recordings could be leveraged to aid implant programming and as supplemental information about neuronal activation during chronic use. For instance, the three semicircular canals could be stimulated in such a pattern to maximize activation of the target canal, while minimizing current spread to other canals, as has been demonstrated in rhesus macaques (Fridman et al., 2010; DaVidovics et al., 2013).

Finally, recordings during continuous stimulation could provide intriguing insights about the correlation between eCAP responses and behavioral responses, such as eye movement responses (Nguyen et al., 2013) or perceptual thresholds (Merfeld et al., 2005a,b). In another recent study, eCAP recordings were used to locate the site of adaptation following vestibular implant stimulation (Phillips et al., 2016). This could prove particularly useful to provide fundamental insights regarding the plasticity of the vestibular pathways in humans in an unprecedented way, that for the time being has only been available to modeling studies (DiGiovanna et al., 2016) as well as animal research (see e.g., Mitchell et al., 2016).

## Conclusions

Electrically eCAPs were recorded in four human subjects instrumented with a vestibulo-cochlear implant. In particular, eCAP recordings for the mixed and trans-canal setup could provide near-term clinical use by helping to reduce undesired current spread. More research is required to better understand the neuronal origin of eCAPs and to use them during chronic stimulation by, for instance, measuring and maximizing activation of target neurons and minimizing activation of non-target neurons. The full potential of vestibular eCAP measurements to provide fundamental contributions to the knowledge of vestibular neurophysiology has yet to be explored.

## Author contributions

TN, NG, RvdB, J-PG, HK, SM, and AP designed the research protocol, which was revised by the remaining authors. NG, J-PG, and RvdB performed the surgeries. Data acquisition and analysis were performed by TN, MR, SC, and AP. All authors participated in the interpretation of the data and the results. TN and AP drafted the manuscript and all other authors revised it. All authors approved of the current version of the manuscript.

### Conflict of interest statement

MED-EL has provided funding for several research projects (including this one). MED-EL has also supported travel expenses for NG, AP, and RvdB for some scientific conferences. SC, MR, and AP currently have a consulting contract with MED-EL. The other authors declare that the research was conducted in the absence of any commercial or financial relationships that could be construed as a potential conflict of interest.
